# Bis[4-amino-3,5-bis­(pyridin-2-yl)-4*H*-1,2,4-triazole-κ^2^ *N* ^1^,*N* ^5^]diaqua­cobalt(II) bis­(perchlorate)

**DOI:** 10.1107/S1600536812038573

**Published:** 2012-09-15

**Authors:** Mi Feng, Yu-Fei Ji, Sheng-Li Liang, Zhi-Liang Liu

**Affiliations:** a College of Chemistry and Chemical Engineering, Inner Mongolia University, Hohhot, People’s Republic of China

## Abstract

In the title structure, [Co(C_12_H_10_N_6_)_2_(H_2_O)_2_](ClO_4_)_2_, the Co^II^ atom lies on an inversion centre and is coordinated in a slightly distorted octa­hedral geometry by four N atoms from two 4-amino-3,5-bis­(pyridin-2-yl)-4*H*-1,2,4-triazole (adpt) ligands in equatorial positions and two O atoms from two water mol­ecules in axial positions. An intra­molecular N—H⋯N inter­action stabilizes the mol­ecular conformation. Inter­molecular N—H⋯O and O—H⋯O inter­actions involving the perchlorate counter-anions extend the monomeric compound into a two-dimensional network parallel to the *bc* plane.

## Related literature

For the synthesis of the adpt ligand, see: Geldard & Lions (1965 ▶). For background to the coordination chemistry of the adpt ligand, see: Meng *et al.* (2009 ▶). For intra­molecular hydrogen bonds in the adpt ligand, see: Kitchen *et al.* (2008 ▶). For other Co(II) coordination compounds with the same ligand, see: Keij *et al.* (1984 ▶); Peng *et al.* (2006 ▶); García-Couceiro *et al.* (2009 ▶); White *et al.* (2010 ▶).
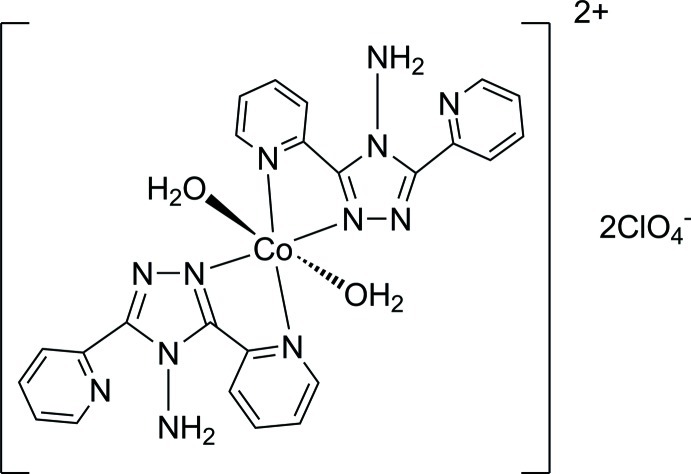


## Experimental

### 

#### Crystal data

[Co(C_12_H_10_N_6_)_2_(H_2_O)_2_](ClO_4_)_2_
*M* *_r_* = 770.38
Monoclinic, 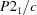

*a* = 8.5839 (17) Å
*b* = 12.950 (3) Å
*c* = 14.975 (5) Å
β = 114.34 (2)°
*V* = 1516.7 (7) Å^3^
*Z* = 2
Mo *K*α radiation
μ = 0.82 mm^−1^
*T* = 293 K
0.04 × 0.03 × 0.01 mm

#### Data collection

Bruker SMART APEX CCD diffractometer
Absorption correction: multi-scan (*SADABS*; Sheldrick, 1996 ▶) *T*_min_ = 0.971, *T*_max_ = 0.992
10155 measured reflections
2681 independent reflections
2336 reflections with *I* > 2σ(*I*)
*R*_int_ = 0.038

#### Refinement

*R*[*F* ^2^ > 2σ(*F* ^2^)] = 0.050
*wR*(*F* ^2^) = 0.121
*S* = 1.07
2681 reflections
223 parameters
H-atom parameters constrained
Δρ_max_ = 0.87 e Å^−3^
Δρ_min_ = −0.50 e Å^−3^

### 

Data collection: *SMART* (Bruker, 2001 ▶); cell refinement: *SAINT* (Bruker, 2001 ▶); data reduction: *SAINT*; program(s) used to solve structure: *SHELXS97* (Sheldrick, 2008 ▶); program(s) used to refine structure: *SHELXL97* (Sheldrick, 2008 ▶); molecular graphics: *DIAMOND* (Brandenburg & Putz, 2006 ▶); software used to prepare material for publication: *publCIF* (Westrip, 2010 ▶).

## Supplementary Material

Crystal structure: contains datablock(s) I, global. DOI: 10.1107/S1600536812038573/wm2664sup1.cif

Structure factors: contains datablock(s) I. DOI: 10.1107/S1600536812038573/wm2664Isup2.hkl

Additional supplementary materials:  crystallographic information; 3D view; checkCIF report

## Figures and Tables

**Table 1:** Hydrogen-bond geometry (Å, °)

*D*—H⋯*A*	*D*—H	H⋯*A*	*D*⋯*A*	*D*—H⋯*A*
N6—H6*B*⋯N5	0.89	2.29	2.897 (4)	125
N6—H6*B*⋯O3^i^	0.89	2.39	2.989 (4)	124
O1—H1*A*⋯O4	0.85	2.16	2.782 (4)	130
O1—H1*B*⋯O2^ii^	0.85	2.22	2.983 (4)	150
O1—H1*B*⋯O5^ii^	0.85	2.58	3.284 (5)	141

Symmetry codes: (i) 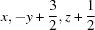; (ii) 
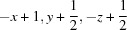
.

**Fig. 1.**
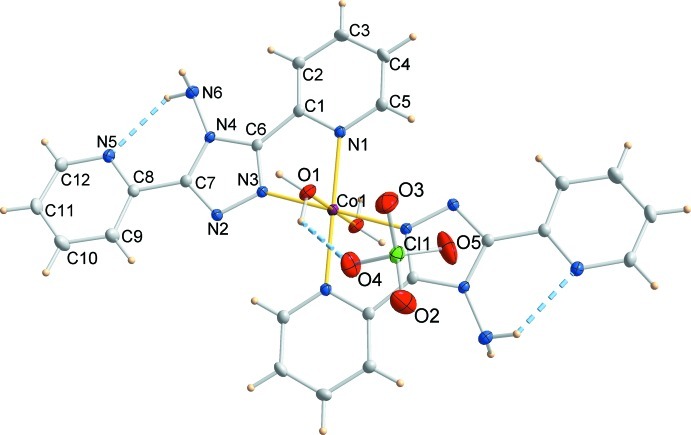
The molecular structure of the title compound, with atom labels and 30% probability displacement ellipsoids for non-H atoms. N—H···N and O—H···O interactions are shown as dashed lines.

**Fig. 2.**
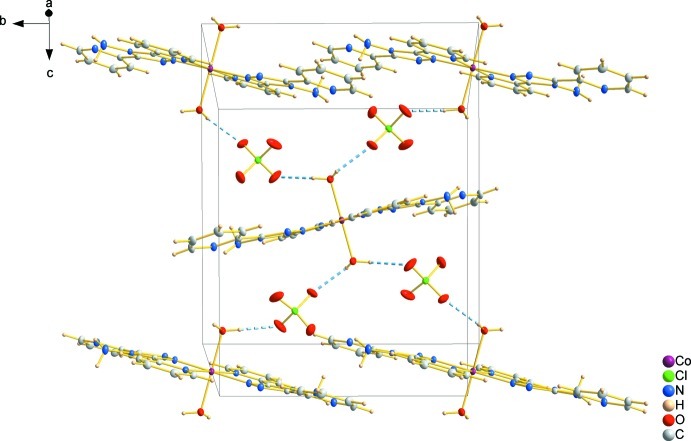
The crystal packing of the title compound. O—H···O interactions are shown as dashed lines.

## supplementary crystallographic information

### Comment

Recently, 4-amine-3,5-di-2-pyridyl-1,2,4-triazol (adpt) has been used as a potential multidentate ligand to generate novel metal-organic complexes due to containing five N coordination sites and three potentially conjugated aromatic rings (Meng *et al.*, 2009). Such complexes with adpt have interesting properties for potential applications in the fields of magnetic materials (Keij *et al.*, 1984). Several Co(II) compounds containing adpt have been reported previously (Keij *et al.*, 1984; Peng *et al.*, 2006; García-Couceiro *et al.*, 2009; White *et al.*, 2010). Herein, the synthesis and crystal structure of the title complex [Co(C_12_H_10_N_6_)_2_(H_2_O)_2_](ClO_4_)_2_, (I), is reported.

As shown in Figure 1, compound (I) consists of one Co(II) atom located on an inversion centre, two adpt ligands, two water molecules and two isolated perchlorate counter anions. The Co(II) is six-coordinated by four N atoms from two adpt ligands and two O atoms from two water molecules, giving a slightly distorted octahedral coordination environment. The equatorial plane is defined by four N atoms from two adpt ligands with a chelate formation, and the axial positions are occupied by two O atoms of water molecules. The dihedral angle between the non-coordinated pyridine ring and the coordinating pyridine ring is 11.94 (16) ° and that between the coordinating pyridine ring and the triazole ring is 6.76 (6)°. In the mononuclear unit, an intramolecular N—H···N hydrogen-bonding interaction between the NH_2_ group attached to the the triazole ring and the non-coordinating N atom of pyridine is observed (Kitchen *et al.*, 2008). Intermolecular N—H···O and O—H···O hydrogen-bonding interactions exist between the amine group and the coordinating water molecules, respectively, with the O atoms of the isolated perchlorate counter anions. In the crystal, the molecular entities are linked by O—H···O hydrogen bonds generating chains along the *b* axis. These chains in turn aggregate into a two-dimensional network parallel to the *bc* plane (Fig. 2).

### Experimental

4-Amino-3,5-bis(pyridin-2-yl)-4H-1,2,4-triazole (abpt, 5mmol) was dissolved in 20 ml mixture solution of water and methanol (1:1, *v*/*v*). Then Co(ClO_4_)_2`_4H_2_O (5 mmol) was added to the above solution. The resulting solution was stirred for 3 h at room temperature. Upon slow evaporation of the solvent, dark red block-shaped crystals formed from the filtrate in a few days. The used 4-amine-3,5-di-2-pyridyl-1,2,4-triazol (adpt) was synthesized according to the previously reported procedure (Geldard & Lions, 1965).

### Refinement

All H atoms were placed in geometrically idealized positions and constrained to ride on their parent atoms with C—H = 0.93 Å and with *U*_iso_(H) = 1.2*U*_eq_(C). H atoms bonded to N and O atoms were located in a difference map and refined with a fixed distance of O—H = 0.85 and N—H = 0.89 Å, and with *U*_iso_(H) = 1.2*U*_eq_(N,O).

### Crystal data

**Table:**

[Co(C_12_H_10_N_6_)_2_(H_2_O)_2_](ClO_4_)_2_	*F*(000) = 786
*M**_r_* = 770.38	*D*_x_ = 1.687 Mg m^−^^3^
Monoclinic, *P*2_1_/*c*	Mo *K*α radiation, λ = 0.71073 Å
Hall symbol: -P 2ybc	Cell parameters from 3601 reflections
*a* = 8.5839 (17) Å	θ = 2.2–27.9°
*b* = 12.950 (3) Å	µ = 0.82 mm^−^^1^
*c* = 14.975 (5) Å	*T* = 293 K
β = 114.34 (2)°	Block, dark red
*V* = 1516.7 (7) Å^3^	0.04 × 0.03 × 0.01 mm
*Z* = 2	

### Data collection

**Table:**

Bruker SMART APEX CCD diffractometer	2681 independent reflections
Radiation source: fine-focus sealed tube	2336 reflections with *I* > 2σ(*I*)
Graphite monochromator	*R*_int_ = 0.038
ω scans	θ_max_ = 25.0°, θ_min_ = 2.2°
Absorption correction: multi-scan (*SADABS*; Sheldrick, 1996)	*h* = −7→10
*T*_min_ = 0.971, *T*_max_ = 0.992	*k* = −15→15
10155 measured reflections	*l* = −17→15

### Refinement

**Table:**

Refinement on *F*^2^	Primary atom site location: structure-invariant direct methods
Least-squares matrix: full	Secondary atom site location: difference Fourier map
*R*[*F*^2^ > 2σ(*F*^2^)] = 0.050	Hydrogen site location: inferred from neighbouring sites
*wR*(*F*^2^) = 0.121	H-atom parameters constrained
*S* = 1.07	*w* = 1/[σ^2^(*F*_o_^2^) + (0.0587*P*)^2^ + 2.3812*P*] where *P* = (*F*_o_^2^ + 2*F*_c_^2^)/3
2681 reflections	(Δ/σ)_max_ < 0.001
223 parameters	Δρ_max_ = 0.87 e Å^−^^3^
0 restraints	Δρ_min_ = −0.50 e Å^−^^3^

### Special details

**Table:**

Geometry. All e.s.d.'s (except the e.s.d. in the dihedral angle between two l.s. planes) are estimated using the full covariance matrix. The cell e.s.d.'s are taken into account individually in the estimation of e.s.d.'s in distances, angles and torsion angles; correlations between e.s.d.'s in cell parameters are only used when they are defined by crystal symmetry. An approximate (isotropic) treatment of cell e.s.d.'s is used for estimating e.s.d.'s involving l.s. planes.
Refinement. Refinement of *F*^2^ against ALL reflections. The weighted *R*-factor *wR* and goodness of fit *S* are based on *F*^2^, conventional *R*-factors *R* are based on *F*, with *F* set to zero for negative *F*^2^. The threshold expression of *F*^2^ > σ(*F*^2^) is used only for calculating *R*-factors(gt) *etc*. and is not relevant to the choice of reflections for refinement. *R*-factors based on *F*^2^ are statistically about twice as large as those based on *F*, and *R*- factors based on ALL data will be even larger.

### Fractional atomic coordinates and isotropic or equivalent isotropic displacement parameters (Å^2^)

**Table:**

	*x*	*y*	*z*	*U*_iso_*/*U*_eq_	
Co1	0.5000	0.5000	0.5000	0.0181 (2)	
Cl1	0.51836 (11)	0.31957 (6)	0.20373 (6)	0.0255 (2)	
N1	0.2655 (3)	0.5819 (2)	0.46508 (18)	0.0182 (6)	
N2	0.7370 (3)	0.6987 (2)	0.59168 (19)	0.0203 (6)	
N3	0.5833 (3)	0.64755 (19)	0.55294 (19)	0.0188 (6)	
N4	0.5287 (3)	0.80947 (19)	0.56599 (18)	0.0165 (6)	
N5	0.7829 (4)	0.9719 (2)	0.6482 (2)	0.0247 (6)	
N6	0.4322 (4)	0.9001 (2)	0.5607 (2)	0.0295 (7)	
H6A	0.3984	0.9278	0.5012	0.044*	
H6B	0.4971	0.9452	0.6054	0.044*	
O1	0.5064 (3)	0.54303 (18)	0.36619 (16)	0.0287 (6)	
H1A	0.5952	0.5177	0.3628	0.043*	
H1B	0.5093	0.6085	0.3630	0.043*	
O2	0.6254 (4)	0.2589 (3)	0.1740 (3)	0.0633 (10)	
O3	0.4116 (4)	0.3823 (2)	0.1213 (2)	0.0555 (9)	
O4	0.6210 (4)	0.3837 (2)	0.2840 (2)	0.0475 (8)	
O5	0.4126 (4)	0.2533 (3)	0.2297 (2)	0.0687 (11)	
C1	0.2804 (4)	0.6823 (2)	0.4928 (2)	0.0181 (7)	
C2	0.1401 (4)	0.7437 (3)	0.4777 (2)	0.0229 (7)	
H2	0.1537	0.8123	0.4977	0.027*	
C3	−0.0217 (4)	0.7004 (3)	0.4318 (3)	0.0273 (8)	
H3	−0.1184	0.7398	0.4211	0.033*	
C4	−0.0381 (4)	0.5988 (3)	0.4024 (2)	0.0261 (8)	
H4	−0.1457	0.5692	0.3704	0.031*	
C5	0.1082 (4)	0.5415 (3)	0.4210 (2)	0.0228 (7)	
H5	0.0969	0.4724	0.4023	0.027*	
C6	0.4598 (4)	0.7148 (2)	0.5385 (2)	0.0171 (7)	
C7	0.7014 (4)	0.7965 (2)	0.5981 (2)	0.0190 (7)	
C8	0.8325 (4)	0.8777 (2)	0.6329 (2)	0.0205 (7)	
C9	0.9991 (4)	0.8547 (3)	0.6470 (3)	0.0285 (8)	
H9	1.0293	0.7879	0.6378	0.034*	
C10	1.1191 (5)	0.9332 (3)	0.6750 (3)	0.0334 (9)	
H10	1.2307	0.9207	0.6828	0.040*	
C11	1.0696 (5)	1.0310 (3)	0.6914 (3)	0.0313 (8)	
H11	1.1477	1.0851	0.7107	0.038*	
C12	0.9036 (5)	1.0464 (3)	0.6786 (3)	0.0287 (8)	
H12	0.8726	1.1116	0.6915	0.034*	

### Atomic displacement parameters (Å^2^)

**Table:**

	*U*^11^	*U*^22^	*U*^33^	*U*^12^	*U*^13^	*U*^23^
Co1	0.0183 (3)	0.0139 (3)	0.0231 (3)	−0.0002 (2)	0.0096 (3)	−0.0014 (2)
Cl1	0.0309 (5)	0.0211 (4)	0.0282 (5)	−0.0011 (3)	0.0160 (4)	−0.0031 (3)
N1	0.0186 (14)	0.0168 (14)	0.0212 (13)	−0.0010 (11)	0.0103 (11)	0.0001 (11)
N2	0.0157 (14)	0.0183 (14)	0.0245 (14)	−0.0012 (11)	0.0058 (12)	−0.0021 (11)
N3	0.0174 (14)	0.0143 (13)	0.0244 (14)	0.0001 (11)	0.0083 (12)	−0.0013 (11)
N4	0.0191 (14)	0.0131 (13)	0.0185 (13)	0.0010 (10)	0.0091 (11)	−0.0003 (10)
N5	0.0233 (15)	0.0187 (14)	0.0297 (16)	−0.0012 (12)	0.0086 (13)	−0.0012 (12)
N6	0.0262 (16)	0.0185 (15)	0.0424 (18)	0.0023 (12)	0.0128 (14)	−0.0011 (13)
O1	0.0389 (15)	0.0206 (12)	0.0312 (13)	0.0066 (11)	0.0191 (12)	0.0034 (10)
O2	0.0474 (19)	0.070 (2)	0.073 (2)	0.0008 (17)	0.0256 (17)	−0.0440 (19)
O3	0.062 (2)	0.0395 (18)	0.0509 (18)	0.0045 (16)	0.0092 (16)	0.0161 (14)
O4	0.0537 (19)	0.0519 (18)	0.0393 (16)	−0.0126 (15)	0.0217 (14)	−0.0229 (14)
O5	0.060 (2)	0.096 (3)	0.0425 (18)	−0.046 (2)	0.0130 (16)	0.0131 (18)
C1	0.0217 (17)	0.0179 (16)	0.0181 (16)	−0.0007 (13)	0.0116 (14)	0.0017 (12)
C2	0.0237 (18)	0.0196 (17)	0.0283 (18)	0.0016 (14)	0.0138 (15)	−0.0002 (14)
C3	0.0205 (18)	0.0293 (19)	0.035 (2)	0.0053 (15)	0.0145 (16)	0.0059 (15)
C4	0.0165 (17)	0.032 (2)	0.0295 (18)	−0.0049 (15)	0.0090 (15)	−0.0010 (15)
C5	0.0240 (18)	0.0206 (17)	0.0268 (17)	−0.0019 (14)	0.0134 (15)	−0.0012 (14)
C6	0.0212 (17)	0.0156 (16)	0.0175 (15)	−0.0007 (13)	0.0109 (13)	0.0007 (12)
C7	0.0218 (17)	0.0181 (16)	0.0193 (16)	−0.0002 (13)	0.0107 (14)	0.0002 (13)
C8	0.0214 (17)	0.0196 (17)	0.0198 (16)	−0.0013 (13)	0.0077 (14)	−0.0007 (13)
C9	0.0248 (19)	0.0256 (19)	0.0339 (19)	−0.0015 (15)	0.0111 (16)	−0.0073 (15)
C10	0.0230 (19)	0.039 (2)	0.038 (2)	−0.0043 (16)	0.0120 (17)	−0.0086 (17)
C11	0.028 (2)	0.029 (2)	0.031 (2)	−0.0115 (16)	0.0065 (16)	−0.0048 (16)
C12	0.029 (2)	0.0191 (18)	0.0338 (19)	−0.0032 (15)	0.0089 (16)	−0.0063 (15)

### Geometric parameters (Å, º)

**Table:**

Co1—N3	2.079 (3)	N6—H6B	0.8901
Co1—N3^i^	2.079 (3)	O1—H1A	0.8500
Co1—O1^i^	2.102 (2)	O1—H1B	0.8500
Co1—O1	2.102 (2)	C1—C2	1.381 (4)
Co1—N1	2.141 (3)	C1—C6	1.466 (4)
Co1—N1^i^	2.141 (3)	C2—C3	1.389 (5)
Cl1—O2	1.413 (3)	C2—H2	0.9300
Cl1—O5	1.416 (3)	C3—C4	1.376 (5)
Cl1—O4	1.428 (3)	C3—H3	0.9300
Cl1—O3	1.446 (3)	C4—C5	1.385 (5)
N1—C5	1.341 (4)	C4—H4	0.9300
N1—C1	1.355 (4)	C5—H5	0.9300
N2—C7	1.316 (4)	C7—C8	1.470 (4)
N2—N3	1.372 (4)	C8—C9	1.389 (5)
N3—C6	1.319 (4)	C9—C10	1.384 (5)
N4—C6	1.350 (4)	C9—H9	0.9300
N4—C7	1.367 (4)	C10—C11	1.390 (5)
N4—N6	1.420 (4)	C10—H10	0.9300
N5—C8	1.342 (4)	C11—C12	1.372 (5)
N5—C12	1.350 (5)	C11—H11	0.9300
N6—H6A	0.8901	C12—H12	0.9300
			
N3—Co1—N3^i^	180.0	H1A—O1—H1B	109.5
N3—Co1—O1^i^	91.15 (10)	N1—C1—C2	122.4 (3)
N3^i^—Co1—O1^i^	88.85 (10)	N1—C1—C6	111.5 (3)
N3—Co1—O1	88.85 (10)	C2—C1—C6	126.1 (3)
N3^i^—Co1—O1	91.15 (10)	C1—C2—C3	118.4 (3)
O1^i^—Co1—O1	180.000 (1)	C1—C2—H2	120.8
N3—Co1—N1	77.24 (10)	C3—C2—H2	120.8
N3^i^—Co1—N1	102.76 (10)	C4—C3—C2	119.6 (3)
O1^i^—Co1—N1	88.45 (9)	C4—C3—H3	120.2
O1—Co1—N1	91.55 (9)	C2—C3—H3	120.2
N3—Co1—N1^i^	102.76 (10)	C3—C4—C5	119.0 (3)
N3^i^—Co1—N1^i^	77.24 (10)	C3—C4—H4	120.5
O1^i^—Co1—N1^i^	91.55 (9)	C5—C4—H4	120.5
O1—Co1—N1^i^	88.45 (9)	N1—C5—C4	122.3 (3)
N1—Co1—N1^i^	180.000 (1)	N1—C5—H5	118.8
O2—Cl1—O5	108.9 (3)	C4—C5—H5	118.8
O2—Cl1—O4	109.47 (19)	N3—C6—N4	109.2 (3)
O5—Cl1—O4	111.4 (2)	N3—C6—C1	120.4 (3)
O2—Cl1—O3	108.0 (2)	N4—C6—C1	130.3 (3)
O5—Cl1—O3	108.8 (2)	N2—C7—N4	110.1 (3)
O4—Cl1—O3	110.2 (2)	N2—C7—C8	123.2 (3)
C5—N1—C1	118.3 (3)	N4—C7—C8	126.7 (3)
C5—N1—Co1	125.6 (2)	N5—C8—C9	123.3 (3)
C1—N1—Co1	116.1 (2)	N5—C8—C7	117.4 (3)
C7—N2—N3	106.5 (3)	C9—C8—C7	119.3 (3)
C6—N3—N2	108.6 (2)	C10—C9—C8	118.7 (3)
C6—N3—Co1	114.6 (2)	C10—C9—H9	120.7
N2—N3—Co1	136.4 (2)	C8—C9—H9	120.7
C6—N4—C7	105.7 (3)	C9—C10—C11	118.7 (3)
C6—N4—N6	124.2 (3)	C9—C10—H10	120.7
C7—N4—N6	130.1 (3)	C11—C10—H10	120.7
C8—N5—C12	117.0 (3)	C12—C11—C10	118.9 (3)
N4—N6—H6A	109.3	C12—C11—H11	120.6
N4—N6—H6B	109.2	C10—C11—H11	120.6
H6A—N6—H6B	109.5	N5—C12—C11	123.5 (3)
Co1—O1—H1A	109.3	N5—C12—H12	118.2
Co1—O1—H1B	109.3	C11—C12—H12	118.2
			
N3—Co1—N1—C5	−179.1 (3)	Co1—N3—C6—N4	−173.37 (18)
N3^i^—Co1—N1—C5	0.9 (3)	N2—N3—C6—C1	176.9 (3)
O1^i^—Co1—N1—C5	−87.5 (3)	Co1—N3—C6—C1	3.0 (4)
O1—Co1—N1—C5	92.5 (3)	C7—N4—C6—N3	0.0 (3)
N3—Co1—N1—C1	−0.9 (2)	N6—N4—C6—N3	−179.7 (3)
N3^i^—Co1—N1—C1	179.1 (2)	C7—N4—C6—C1	−175.8 (3)
O1^i^—Co1—N1—C1	90.7 (2)	N6—N4—C6—C1	4.4 (5)
O1—Co1—N1—C1	−89.3 (2)	N1—C1—C6—N3	−3.6 (4)
C7—N2—N3—C6	−1.0 (3)	C2—C1—C6—N3	176.9 (3)
C7—N2—N3—Co1	171.1 (2)	N1—C1—C6—N4	171.8 (3)
O1^i^—Co1—N3—C6	−89.3 (2)	C2—C1—C6—N4	−7.7 (5)
O1—Co1—N3—C6	90.7 (2)	N3—N2—C7—N4	1.0 (3)
N1—Co1—N3—C6	−1.1 (2)	N3—N2—C7—C8	−177.6 (3)
N1^i^—Co1—N3—C6	178.9 (2)	C6—N4—C7—N2	−0.6 (3)
O1^i^—Co1—N3—N2	99.0 (3)	N6—N4—C7—N2	179.1 (3)
O1—Co1—N3—N2	−81.0 (3)	C6—N4—C7—C8	177.8 (3)
N1—Co1—N3—N2	−172.8 (3)	N6—N4—C7—C8	−2.4 (5)
N1^i^—Co1—N3—N2	7.2 (3)	C12—N5—C8—C9	0.2 (5)
C5—N1—C1—C2	0.3 (4)	C12—N5—C8—C7	−178.9 (3)
Co1—N1—C1—C2	−178.0 (2)	N2—C7—C8—N5	−171.5 (3)
C5—N1—C1—C6	−179.2 (3)	N4—C7—C8—N5	10.2 (5)
Co1—N1—C1—C6	2.5 (3)	N2—C7—C8—C9	9.4 (5)
N1—C1—C2—C3	−0.4 (5)	N4—C7—C8—C9	−169.0 (3)
C6—C1—C2—C3	179.1 (3)	N5—C8—C9—C10	−2.3 (5)
C1—C2—C3—C4	−0.4 (5)	C7—C8—C9—C10	176.8 (3)
C2—C3—C4—C5	1.3 (5)	C8—C9—C10—C11	2.3 (5)
C1—N1—C5—C4	0.6 (5)	C9—C10—C11—C12	−0.4 (5)
Co1—N1—C5—C4	178.8 (2)	C8—N5—C12—C11	1.9 (5)
C3—C4—C5—N1	−1.4 (5)	C10—C11—C12—N5	−1.8 (6)
N2—N3—C6—N4	0.6 (3)		

Symmetry code: (i) −*x*+1, −*y*+1, −*z*+1.

### Hydrogen-bond geometry (Å, º)

**Table:**

*D*—H···*A*	*D*—H	H···*A*	*D*···*A*	*D*—H···*A*
N6—H6*B*···N5	0.89	2.29	2.897 (4)	125
N6—H6*B*···O3^ii^	0.89	2.39	2.989 (4)	124
O1—H1*A*···O4	0.85	2.16	2.782 (4)	130
O1—H1*B*···O2^iii^	0.85	2.22	2.983 (4)	150
O1—H1*B*···O5^iii^	0.85	2.58	3.284 (5)	141

Symmetry codes: (ii) *x*, −*y*+3/2, *z*+1/2; (iii) −*x*+1, *y*+1/2, −*z*+1/2.
